# Thalidomide-induced serious RR interval prolongation (longest interval >5.0 s) in multiple myeloma patient with rectal cancer: A case report

**DOI:** 10.1515/med-2020-0136

**Published:** 2020-06-11

**Authors:** Li Huang, Yuemin Kuang, Zhiyong Jiang, Yan Zhu, Xinguo Luo, Fangjing Shi, Shanshan Hu, Xinfang Gao

**Affiliations:** Department of Hematology, Jinhua People’s Hospital, No. 228 Xinhua Street, Jinhua, Zhejiang, 32100, China

**Keywords:** RR interval prolongation, multiple myeloma, rectal cancer

## Abstract

Primary secondary tumor increased recently with the use of immunomodulatory drugs in patients with multiple myeloma (MM). However, MM with prior diagnosis of primary secondary tumor is relatively rare. In this study, we reported an MM patient with prior diagnosis of rectal cancer. In brief, an 85-year-old man was first diagnosed with rectal cancer. Given the age, heart failure and small-cell hypochromic anemia (hemoglobin level: 54 g/L), rectal cancer resection was not advised and symptomatic treatments were performed (including sufficient iron supplementation). Eight months later, the patient was diagnosed with MM due to worsening anemia. Anemia and heart failure were corrected after three cycles of treatment with thalidomide, dexamethasone and capecitabine. Radical resection of rectal carcinoma (Hartmann) was finally performed due to acute abdominal distension. Meanwhile, RR interval prolongation (longest interval >5.0 s) and atrial fibrillation occurred in the fifth cycle treatment. One month after discontinuation of thalidomide, RR interval returned to normal range, while atrial fibrillation developed into persistent atrial fibrillation.

## 1 Introduction

Survival of patients with multiple myeloma (MM) has improved dramatically since the introduction of stem-cell transplantation and new agents such as immunomodulatory drugs (thalidomide and lenalidomide) and proteasome inhibitors [1]. However, relapse remains frequent even in patients with favorable genetic profiles who undergo intensive induction therapy and tandem stem-cell transplantation [2]. Relapse is due to the persistence of malignant plasma cells, and as a consequence long-term maintenance strategies to control residual disease have been assessed [3]. Recent studies showed that maintenance therapies (especially immunomodulatory drugs) increased the risk of second malignancies [4,5]. However, MM with prior diagnosis of rectal cancer is relatively rare. Meanwhile, the treatment of MM can also increase cardiovascular complications [6]. Immunomodulatory drug-related cardiovascular events are mainly vein and arterial thromboses [6]. It can also cause bradycardia and even total atrioventricular block [7]. In this article, we reported a case of thalidomide-induced serious RR interval prolongation (longest interval >5.0 s) in MM patient with prior diagnosis of rectal cancer.

## 2 Case report

**Figure 1:**
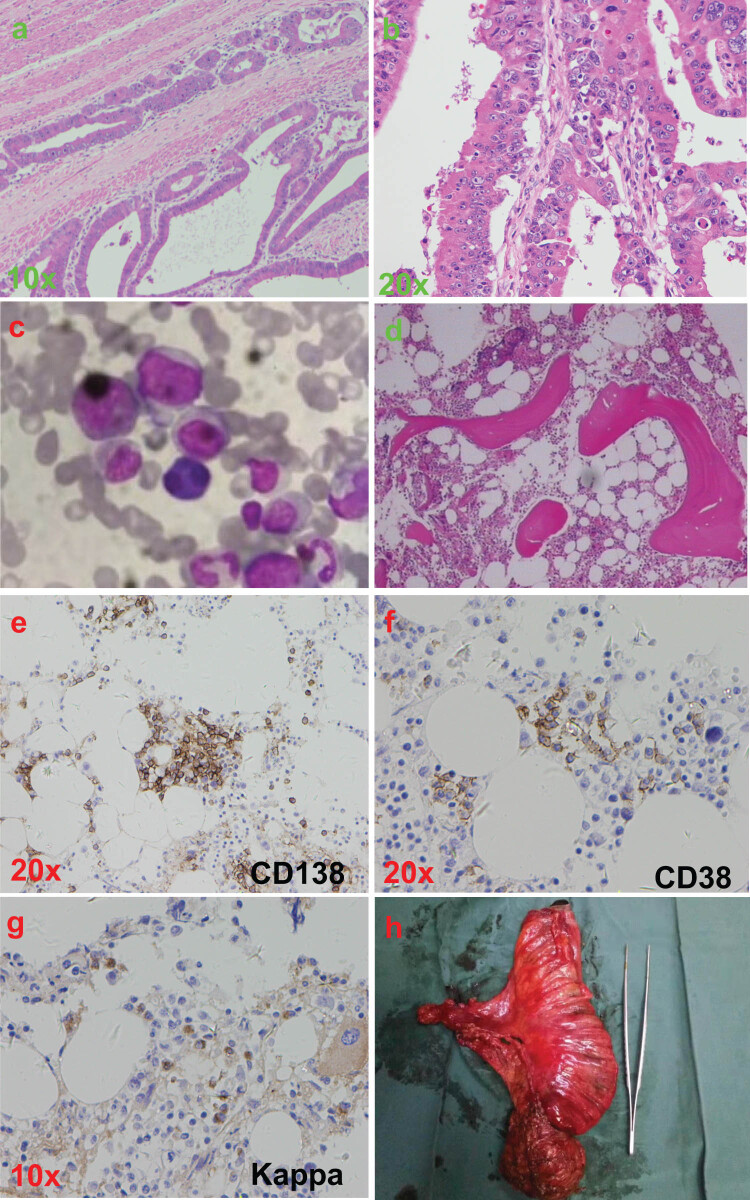
Clinical and histopathological appearances. (a and b) HE staining results from thickened rectum showing adenocarcinoma. (c) Bone marrow cytology results showing increased plasmocyte. (d–g) Pathological and immunohistochemical results of bone marrow showing CD138 (+), CD38 (+), CD56 (partial +), Kappa (+), myelodysplastic activity and increased plasmocyte. (h) Excised rectal cancer.

An 85-year-old man presented in the Department of Cardiology of Jinhua People’s Hospital because of chest distress for 1 month (March 26, 2017). The percussion examination of chest showed bilateral dullness. Blood test showed a white blood cell (WBC) level of 4.58 × 10^9^/L, a hemoglobin (HGB) level of 54 g/L, a mean corpuscular hemoglobin (MCH) level of 20.1 pg, a mean corpuscular hemoglobin concentration (MCHC) level of 295 g/L, a platelet (PLT) level of 223 × 10^9^/L, a ferroprotein level of 9.11 ng/mL, a carcinoembryonic antigen (CEA) level of 136.72 ng/mL, and fecal occult blood test (−). Computed tomography results of chest and abdomen showed moderate pleural effusion on both sides, slightly thickened wall of rectum and rectal obstruction. Pathological results of thickened rectum showed adenocarcinoma (Figure 1a and b). Biochemical results of pleural effusion indicated leaking fluid. The patient was diagnosed with rectal cancer, heart failure, small-cell hypochromic anemia, and diabetes. After consultation with cardiologists, respiratory physician and anesthesiologists, rectal cancer resection was not performed. The patient was discharged after blood transfusion, iron supplementation, drainage of pleural effusion, treatment of heart failure and control of blood glucose. Iron supplementation was continued after being discharged from the hospital.

Eight months later (December 9, 2017), the patient was hospitalized again due to aggravated chest tightness. Blood test showed a WBC level of 9.49 × 10^9^/L, an HGB level of 30 g/L, a PLT level of 336 × 10^9^/L, a CEA level of 185.83 ng/mL, an immunoglobulin (Ig) A level of 11.1 g/L and an IgM level of 0.37 g/L. Monoclonal IgA-ƙ and light chain ƙ were detected in blood and urine, respectively. Bone marrow cytology results showed increased plasmocyte (12.3% of karyocyte; Figure 1c). Pathological and immunohistochemical results of bone marrow showed CD138 (+), CD38 (+), CD56 (partial +), Kappa (+), Lambda (−), CD3 (−), CD19 (−), CD20 (−), myelodysplastic activity and increased plasmocyte (20–30% of karyocyte; Figure 1d–g). The patient was diagnosed with MM (IgA-k DS IIIA/ISS IIA). He was treated with thalidomide (100 mg, q.d., 4 weeks), dexamethasone (20 mg, q.w.d., 4 weeks) and capecitabine (1.5 g, bid, 2 weeks) for three cycles.

The patient complained of abdominal distension on March 19, 2018. Abdominal radiography results showed incomplete intestinal obstruction. Blood test showed a WBC level of 2.82 × 10^9^/L, a neutrophil level of 2.09 × 10^9^/L, an HGB level of 96 g/L, a PLT level of 136 × 10^9^/L, an IgA level of 2.21 g/L and an IgG level of 5 g/L. After consultation with cardiologists, anesthesiologists and oncologist, radical resection of rectal carcinoma (Hartmann) was performed (Figure 1h). Immunohistochemical results of tumor showed LRP (2+), MRP (2+), Ts (−), TOP II-α (1+), β-Catenin (+), CDX2 (+), C-erbB-2 (−), CK20 (+), EGFR (−), EMA (+) and ER (−), P53 (3+) and PR (−), Ki67 (+15%). Pathological results of tumor showed constrictive moderately differentiated adenocarcinoma (T_3_N_0_M_0_, 3 × 2.5 × 1.5 cm).

**Figure 2:**
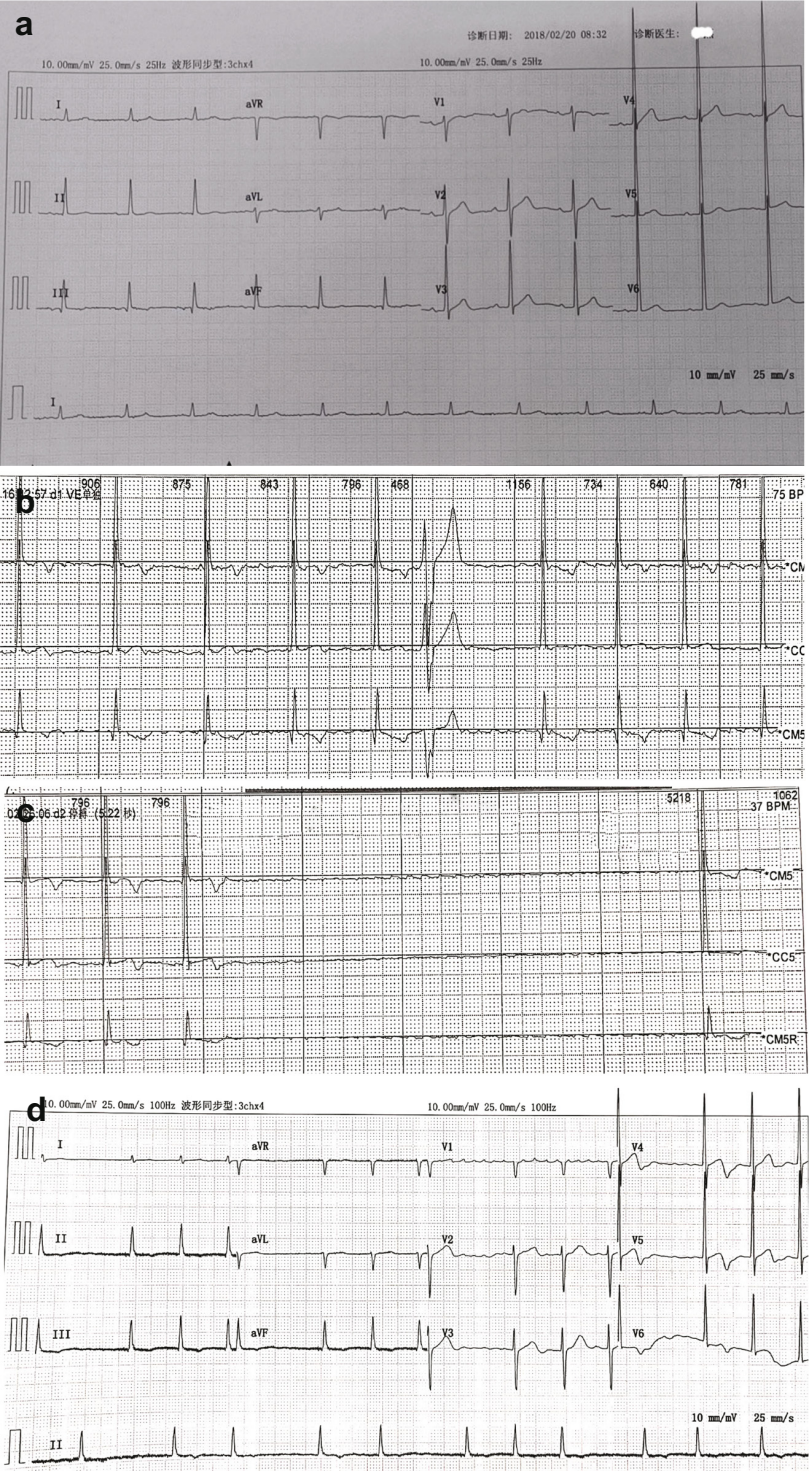
ECG of patient during treatment. (a) Normal ECG before atrial fibrillation and RR interval prolongation; (b) the first holt result showing atrial fibrillation occurring during thalidomide treatment; (c) the first holt result showing serious RR interval prolongation (RR interval = 5.2 s) during thalidomide treatment; and (d) atrial fibrillation did not disappear after withdrawal.

Another two cycles of thalidomide, dexamethasone and capecitabine treatment were started on April 20 and May 12, 2018. Electrocardiogram (ECG) monitor results showed bradyarrhythmia (<40 beats/min) on May 19, 2018. The hotter results showed atrial fibrillation with slow ventricular rate, RR interval prolongation (>2 s having 2,283 times, >3 s having 30 times, the longest RR interval being 5.2 s) and ventricular ectopic beat (Figure 2b and c). Evidence of structural cardiac disease was excluded by cardiac ultrasonography. Cardiologists suggested to install a cardiac pacemaker but the patient refused. Thalidomide was withdrawn because of slow ventricular rate, RR interval prolongation and a potential risk of cardiovascular events. The second hotter results before the sixth treatment showed atrial fibrillation, RR interval prolongation (>2 s having 77 times) and ventricular ectopic beat (June 8, 2018). Considering the cardiotoxicity of thalidomide, patient was treated with dexamethasone (20 mg, q.w.d., 4 weeks) and bortezomib (1.65 mg, q.w.d., 4 weeks). One month after discontinuation of thalidomide, RR interval returned to normal range, while atrial fibrillation did not disappear (Figure 2d). After that, patient suffered bortezomib+dexamethasone (PD) and isazolam citrate+dexamethasone (ID) therapies for several cycles and was in complete remission. Written informed consent was obtained from the patient for publication and this case report was approved by the ethics committee of Jinhua People's Hospital according to tenets of the Declaration of Helsinki.

## 3 Discussion

Recent studies showed that MM patients had a higher risk of developing second malignancy than the general population [8,9]. Mailankody et al. showed that MM patients had an 11-fold increased risk of developing acute myeloid leukemia and myelodysplastic syndromes and a twofold increased risk of developing nonmelanoma skin cancer [8]. However, the risk of colorectal cancer was relatively low. Hasskarl et al. showed that the second solid tumor mostly was diagnosed prior to the MM diagnosis (53%), whereas 20% developed after the myeloma [10]. The median time intervals between MM and second colorectal cancer were 61 (before) and 52 (after) months. The MM patient with synchronous colorectal cancer is rare. In this case, the diagnosis of rectal cancer was prior to MM. However, the time interval between rectal cancer and MM was 8 months, which was obviously shorter than 61 months. Given the severe anemia, negative fecal occult blood test, sufficient iron supplementation and continuously decreased HGB level at first admission and rapidly increased HGB level after MM treatment, we thought that MM may be missed to be diagnosed at first admission. This case reminded that hematologic neoplasms should be considered in solid cancer patients with severe anemia when iron supplementation was ineffective.

Previous study showed that thalidomide-related cardiovascular events were mainly vein and arterial thromboses [6]. Cardiotoxicity caused due to thalidomide was relatively rare. Only a few articles reported that thalidomide could cause bradycardia, total atrioventricular block and coronary artery spasm [7,11]. In this study, we showed that atrial fibrillation and serious RR interval prolongation (longest interval >5.0 s) occurred after four cycles of treatment. The RR interval gradually returned to normal range after discontinuation, indicating that RR interval prolongation was induced by thalidomide. However, atrial fibrillation did not disappear after withdrawal. It was unclear whether thalidomide induced atrial fibrillation. Given normal ECG before treatment and atrial fibrillation accompanying with RR interval prolongation, we considered that atrial fibrillation may also be induced by thalidomide. The potential mechanism of thalidomide-induced RR interval prolongation was unclear. The inward current *I*(*f*) is regulated by the NO signaling pathway. Several studies have shown that thalidomide inhibits the NO signaling pathway by reducing the activity of soluble guanylyl cyclase [12,13]. Disordered inward current *I*(*f*) caused by thalidomide may be the reason for RR interval prolongation.

## 4 Conclusion

Hematologic neoplasms, especially MM, should be considered in solid cancer patients with severe anemia when iron supplementation was ineffective. Thalidomide could induce serious RR interval prolongation.

## Abbreviations

CEA: carcinoembryonic antigen
ECG: electrocardiogram
HGB: hemoglobin
Ig: immunoglobulin
MCH: mean corpuscular hemoglobin
MCHC: mean corpuscular hemoglobin concentration
MM: multiple myeloma
PLT: platelet
WBC: white blood cell

## References

[1] Pratt G. Lenalidomide and second malignancies in myeloma patients. Lancet Oncol. 2014;15(3):253–4. 10.1016/S1470-2045(14)70001-4 24525201

[2] Jones JR, Cairns DA, Gregory WM, Collett C, Pawlyn C, Sigsworth R, et al. Second malignancies in the context of lenalidomide treatment: an analysis of 2732 myeloma patients enrolled to the Myeloma XI trial. Blood Cancer J. 2016;6(12):e506. 10.1038/bcj.2016.114 PMC5223149 27935580

[3] Rawstron AC, Child JA, de Tute RM, Davies FE, Gregory WM, Bell SE, et al. Minimal residual disease assessed by multiparameter flow cytometry in multiple myeloma: impact on outcome in the Medical Research Council Myeloma IX Study. J Clin Oncol. 2013;31:2540–7. 10.1200/JCO.2012.46.2119 23733781

[4] McCarthy PL, Owzar K, Hofmeister CC, Hurd DD, Hassoun H, Richardson PG, et al. Lenalidomide after stem-cell transplantation for multiple myeloma. N Engl J Med. 2012;366(19):1770–81. 10.1056/NEJMoa1114083 PMC3744390 22571201

[5] Landgren O, Thomas A, Mailankody S. Myeloma and second primary cancers. N Engl J Med. 2011;365(23):2241–2. 10.1056/NEJMc1111010 PMC7213755 22150057

[6] Lee DH, Fradley MG. Cardiovascular complications of multiple myeloma treatment: evaluation, management, and prevention. Curr Treat Options Cardiovasc Med. 2018;20(3):19. 10.1007/s11936-018-0618-y 29508087

[7] Liu M, Lin X, Wang L, He Y, Chen M, Mao R. Thalidomide-induced sinus bradycardia in Crohn’s disease: case report and literature review. J Int Med Res. 2019;47(5):2228–33. 10.1177/0300060519833293 PMC6567770 30832535

[8] Mailankody S, Pfeiffer RM, Kristinsson SY, Korde N, Bjorkholm M, Goldin LR, et al. Risk of acute myeloid leukemia and myelodysplastic syndromes after multiple myeloma and its precursor disease (MGUS). Blood. 2011;118(15):4086–92. 10.1182/blood-2011-05-355743 PMC3204729 21795746

[9] Jonsdottir G, Lund SH, Björkholm M, Turesson I, Hultcrantz M, Porwit A, et al. The impact of prior malignancies on second malignancies and survival in MM patients: a population-based study. Blood Adv. 2017;1(25):2392–8. 10.1182/bloodadvances.2017007930 PMC5729617 29296889

[10] Hasskarl J, Ihorst G, De Pasquale D, Schröttner P, Zerweck A, Wäsch R, et al. Association of multiple myeloma with different neoplasms: systematic analysis in consecutive patients with myeloma. Leuk Lymphoma. 2011;52(2):247–59. 10.3109/10428194.2010.529207 21054148

[11] Zhang S, Yang J, Jin X, Zhang S. Myocardial infarction, symptomatic third degree atrioventricular block and pulmonary embolism caused by thalidomide: a case report. BMC Cardiovasc Disord. 2015;15:173. 10.1186/s12872-015-0164-4 PMC4683955 26681197

[12] Majumder S, Rajaram M, Muley A, Reddy HS, Tamilarasan KP, Kolluru GK, et al. Thalidomide attenuates nitric oxide-driven angiogenesis by interacting with soluble guanylyl cyclase. Br J Pharmacol. 2009;158:1720–34. 10.1111/j.1476-5381.2009.00446.x PMC2801213 19912234

[13] Tamilarasan KP, Kolluru GK, Rajaram M, Indhumathy M, Saranya R, Chatterjee S. Thalidomide attenuates nitric oxide mediated angiogenesis by blocking migration of endothelial cells. BMC Cell Biol. 2006;7:17. 10.1186/1471-2121-7-17 PMC1456963 16584574

